# Transstomal Small Bowel Evisceration after Colonic Perforation Secondary to Ischemic Colitis

**DOI:** 10.1155/2012/560683

**Published:** 2012-09-17

**Authors:** Ali Guner, Izzettin Kahraman, Omer Faruk Ozkan, Adem Aktas, Can Kece

**Affiliations:** Department of General Surgery, Trabzon Numune Training and Research Hospital, 61040 Trabzon, Turkey

## Abstract

Intestinal stomas are commonly used in a temporary or permanent fashion in gastrointestinal surgeries. The complication rate of stomas has been reported to vary between 23 and 50%. There is only one case in the literature involving transstomal small bowel evisceration following colonic perforation. In this paper, we aimed to present a patient with a perforated colon secondary to ischemic colitis, which resulted in small bowel evisceration through this perforation site.

## 1. Introduction

Intestinal stomas are commonly used in gastrointestinal surgeries. Common complications of stomas on the abdominal wall include herniation, retraction, stenosis, necrosis, and prolapse. There is only one case in the literature involving transstomal small bowel evisceration following colonic perforation. However, this is the first report after perforation due to ischemic colitis. In this paper, we aimed to present a patient with a perforated colon secondary to ischemic colitis, which resulted in small bowel evisceration through this perforation site.

## 2. Case Presentation

A 76-year-old man with an end colostomy was admitted to the emergency department due to evisceration of the small bowel through the stoma. The patient had received surgery by Hartmann procedure (sigmoid colon resection and end colostomy) 11 months previously because of sigmoid colon necrosis, and he had been discharged 10 days after the operation without any complications. The histopathologic material obtained from the patient had been found to be consistent with ischemic gangrenous colitis. Clinical history of the patient revealed diabetes mellitus and smoking, but he mentioned no symptoms for this period until the abrupt prolapse of the intestines through the intestinal stoma during walking.

**Figure 1:**
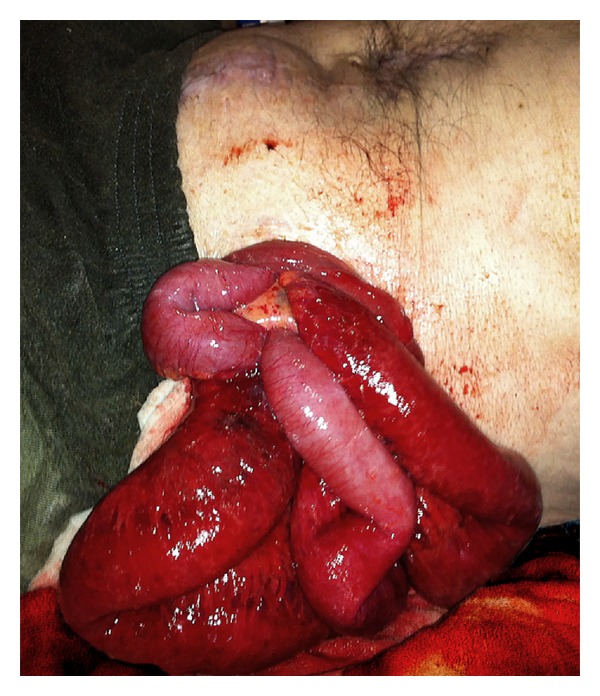
The view of eviscerated small bowels through the colostomy in the emergency department.

**Figure 2:**
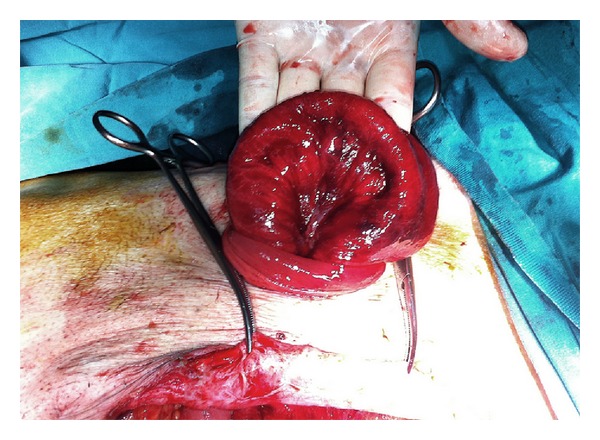
The view of small bowels after partial reduction at laparotomy.

**Figure 3:**
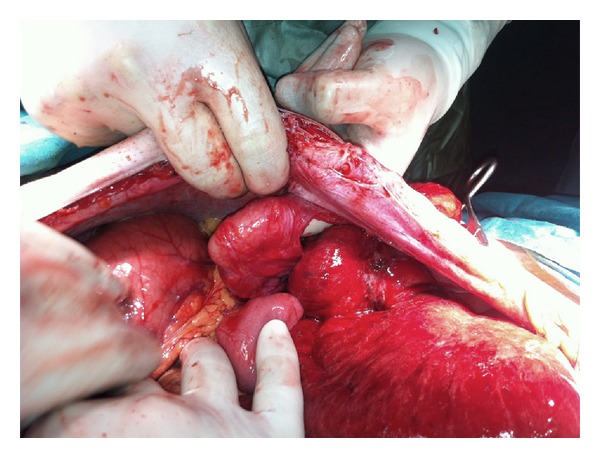
Perforation site of the colon.

The initial examination revealed that the small bowel loops were eviscerated through the stoma, and the intestinal loops were ischemic and edematous while also being irreducible into the abdomen (Figure 1). The patient was operated immediately by a midline laparotomy (Figure 2). Exploration revealed a perforation site 10 cm proximal to the colostomy opening and evisceration of the small bowel loops into the colonic lumen via this hole (Figure 3). Following the reduction of the small bowel loops into the abdomen, left colon was resected in a way to include the perforated site and a new end colostomy was performed with a segment having a normal blood supply. The small bowel received no resection since it had no necrosis. The postoperative course was uneventful and the patient was discharged on the 7th postoperative day. The histopathological examination of the resected colonic segment showed diffuse ischemia and perforation secondary to ischemic colitis. 

## 3. Discussion

Common complications of stomas on the abdominal wall include herniation, retraction, stenosis, necrosis, and prolapse [1]. Complication rates vary depending on the involved intestinal segment, characteristics of the stoma (loop or end stoma), emergency or elective nature of the procedure, and follow-up time. Recent studies report overall risk of stoma complication as 23.5% to 50.5% [2].

In the literature, there is only one case of transstomal small bowel evisceration secondary to events such as acute intestinal obstruction, sudden increase of abdominal pressure, and prolapsing distal colonic lumen [3]. Moreover, despite case reports involving spontaneous rectosigmoid perforation with transanal evisceration secondary to rectal and uterine prolapse, and transvaginal evisceration after hysterectomy, our study is the first case report including a colonic perforation due to ischemic colitis, resulting in small bowel evisceration [4, 5]. 

Ischemic colitis is the most common form of ischemic injury to the gastrointestinal tract. This disease has various clinical subtypes which can present in a wide range of pathologies from transient segmental colopathy to fulminant gangrenous colitis [6]. The patient had a history of surgery performed one year ago due to gangrenous colitis secondary to ischemia, and the following recurrence of ischemia with a more limited nature resulted in local perforation. Because of the strangulation risk of the eviscerated intestinal loops, early surgery bears great importance. Although the perforation site can be managed by debridement and primary repair, segmental resection and a new end colostomy should be preferred due to the underlying ischemic pathology. One should always bear in mind that future abdominal pathologies may present with complications associated with this disease.
